# Individual phenotypic variability in the behaviour of an aggregative riverine fish is structured along a reactive-proactive axis

**DOI:** 10.1371/journal.pone.0312187

**Published:** 2024-11-20

**Authors:** Fatima Amat-Trigo, Demetra Andreou, Phillipa K. Gillingham, J. Robert Britton

**Affiliations:** Department of Life and Environmental Sciences, Bournemouth University, Poole, Dorset, United Kingdom; PSTU: Patuakhali Science and Technology University, BANGLADESH

## Abstract

High phenotypic diversity should provide populations with resilience to environmental change by increasing their capacity to respond to changing conditions. The aim of this study was to identify whether there is consistency in individual behaviours on a reactive-proactive axis in European barbel *Barbus barbus* ("barbel"), a riverine and aggregatory fish that expresses individual differences in its behaviours in nature. This was tested using three sequential experiments in *ex-situ* conditions that required individuals to leave a shelter and then explore new habitats (‘open-field test’), respond to social stimuli (‘mirror-image stimulation test’) and forage (‘foraging behaviour test’; assessing exploratory traits). Each suite of experiments was replicated three times per individual (46 hours minimum time between replicates). There was high variability in behaviours both within and among individuals. The most repeatable behaviours were latency to exit the shelter, active time in the shelter, and the number of food items consumed. Principal component scores did, however, indicate a range of consistent behavioural phenotypes across the individuals, distributing them along a reactive-proactive axis in which most of individuals were more reactive phenotypes (shyer, less exploratory, less social). These results suggest that within controlled conditions, there is considerable phenotypic diversity among individuals in their behaviours, suggesting their populations will have some adaptive capacity to environmental change.

## Introduction

Intra-specific phenotypic variability can strongly affect community structure and ecosystem functioning [1]. This phenotypic variability also extends to animal behaviours, a field of organismal biology which has been increasing in popularity since the 2000s [2]. Work in this field has increasingly identified among-individual behavioural differences (e.g. in sociability, exploration and activity) that are consistent over time and in different situations. This existence of consistent individual behavioural variation within populations has also been apparent over a wide range of species and taxa (e.g. [3]). This behavioural variation can be both among-individuals, where the behaviours of individuals differ from one another, and within-individuals, which describes how consistent those behaviours are within the same individual [2]. In addition, different behaviours have been observed that covary and are consistent among individuals of the same population where, for example, more aggressive individuals tend to also express more exploratory behaviours. Where behaviours are correlated and consistent, they are referred to as behavioural syndromes [4, 5], but with this recently also being referred to as `among-individual correlation´ [2]. Similarly, animal personality refers to this consistent variation in behaviour among individuals and, when correlated across individuals at the population level, can be referred to as behavioural syndromes [5].

Descriptions of behavioural variability among individuals include the bold-shy continuum, where individuals demonstrate consistency in their differences in risk-prone behaviours according to trade-offs between, for example, foraging benefits and predation risks [6–8]. Indeed, this behavioural syndrome is often seen in predator presence, but it can also vary under different social contexts [9, 10]. Differences in among-individual behaviours are important as they can influence individual survival and fitness [11], so have important ecological and evolutionary implications [2, 5, 12, 13]. The proactive-reactive axis is observed in many vertebrate species and whilst it frequently involves the bold-shy continuum, other behaviours (e.g., exploratory and aggressive behaviours) also help to describe this behavioural syndrome. Proactive individuals are usually bolder, more exploratory and aggressive, and are more likely to maintain their behaviours as conditions change [14]. These individuals also generally have more resilient stress coping mechanisms than reactive individuals [15].

Methods to measure the consistency of individual behaviours include assessments of the within-individual repeatability of behaviours over time and across contexts [16]. Several studies have shown that many physiological and behavioural traits have some degree of repeatability within individuals, but that this repeatability can be influenced by different factors such as life stage, sex, populations and environmental conditions [3, 16, 17]. In addition, there is within-individual ’behavioural plasticity’, which suggests that the same individual can have a range of responses to a given situation. This within-individual behavioural plasticity can also be influenced by various factors, such as environmental changes, changes in physiological traits, and/ or by learning processes [18, 19].

Studies on among-individual behavioural correlations (i.e. personality axes) have focused mainly on species with social traits, such as parental care or dominance structures (e.g. [5, 20]), with fewer studies on aggregative species (‘social species’) that show little or no dominance. In social species, selection acts at both individual and group levels, and where bold and social individuals interact, foraging benefits can be apparent [21]. Moreover, the position of individuals on personality continuums and within behavioural syndromes can also potentially explain differences in the spatial behavioural traits of populations [22], with proactive individuals expected to explore and disperse further than reactive ones [23].

The European barbel *Barbus barbus* (L. 1758) (‘barbel’) is a riverine fish that is both aggregative and relatively vagile [24, 25]. The probability of an individual moving from one locality to another between consecutive days can be over 50% during spawning periods [26, 27], although the fidelity of individuals to specific activity areas increases post-spawning [26]. *Barbus barbus* populations also demonstrate considerable among-individual differences in vagility, with populations comprising of ‘resident’ and ‘mobile’ fish [25, 28]. For example, in the River Severn, Western England, an initial study indicated that 86% of 531 tagged fish remained within 5 km of their release point, with some barely moving at all, while the remaining fish were recorded up to 34 km away [29]. A later study in the same river indicated that 83% had home ranges below 5.5 km, but with some individuals having home ranges over 12 km [28]. Whether these differences in individual movements and home ranges are associated with behavioural syndromes is currently unclear, with an absence of information on the extent of the variability between personality traits and behaviours of individual *B*. *barbus*.

The aim here was to test the extent to which *B*. *barbus* exhibit strong and consistent individual differences in behaviours, using the proactive-reactive axis as the basis for testing and, if this axis is apparent, determine which of the measured behaviours is most influential in defining it. Experiments were completed in *ex-situ* conditions across a range of behavioural contexts that describe important aspects of fish behaviour [30]: exploration of new habitats (‘open-field test’); sociability of individuals (‘mirror-image stimulation test’); and risk-taking of individuals (‘foraging behaviour test’). We predict that across the three contexts, individual behaviours are correlated and structured into a proactive-reactive syndrome, where proactive *B*. *barbus* are more exploratory, social and bolder, with these behavioural correlates being highly repeatable within individuals. This prediction was developed through previous studies in cyprinid fishes (including barbel species) that have detected individual behaviours being on a proactive-reactive axis [22] and where the strongest determining behaviours in the development of this axis are highly repeatable [14]. Throughout the paper, we refer mainly to the pro-active/ reactive axis, on which boldness/ shyness is a specific expressed behaviour, but that also includes exploratory behaviours and activity. Our prediction is based on identifying the extent of phenotypic diversity across individuals, where highly repeatable behaviours within-individuals and high behavioural variation among individuals would indicate high phenotypic diversity in the population. Should the fish show high within-individual behaviour with less behavioural variation among individuals then this would indicate that the individual fish are expressing high phenotypic plasticity to the experimental conditions.

## Materials and methods

### Overview of the experimental protocol

Fish used in the experiments were hatchery-reared, rather than wild fish, as this ensured fish were size-matched (6 to 8 cm fork length). The hatchery *B*. *barbus* were also produced from the same brood-stock and all experienced very similar rearing conditions (pond rearing on a mix of natural and formulated food). Once in the laboratory, the fish were acclimated in two groups (n = 24 per group) for 30 days in 100 L aquaria at 17°C under 16:8 h light-dark regime. Feeding was on a maintenance diet (approximately 1 to 1.5% body weight of formulated food per day). At the beginning of the acclimation period, all fish were implanted with a 7 mm passive integrated transponder (PIT) tag (Loligo Systems, Viborg, Denmark) to enable their subsequent individual identification during experiments, and weighed (to 0.1g, OHAUS Pioneer® PX323/E). Following immersion in an anaesthetic bath (Tricaine methanesulfonate, MS222), the PIT tags were inserted into the fish by making a small (<3-mm) incision in the abdominal cavity, behind the left pelvic fin and the abdominal midline. After recovery, the fish were acclimated in the holding aquaria at least two months before to start the experiments in order to ensure the fish were settled in their new environment and that physical recovery had completed from PIT tag implantation (so not to affect any aspect of individual behaviour). Previous studies have demonstrated that PIT tagging does not affect the survival, growth or swimming behaviour in barbel species [31, 32]. The behavioural experiments were then carried out between June and August 2021.

**Fig 1:**
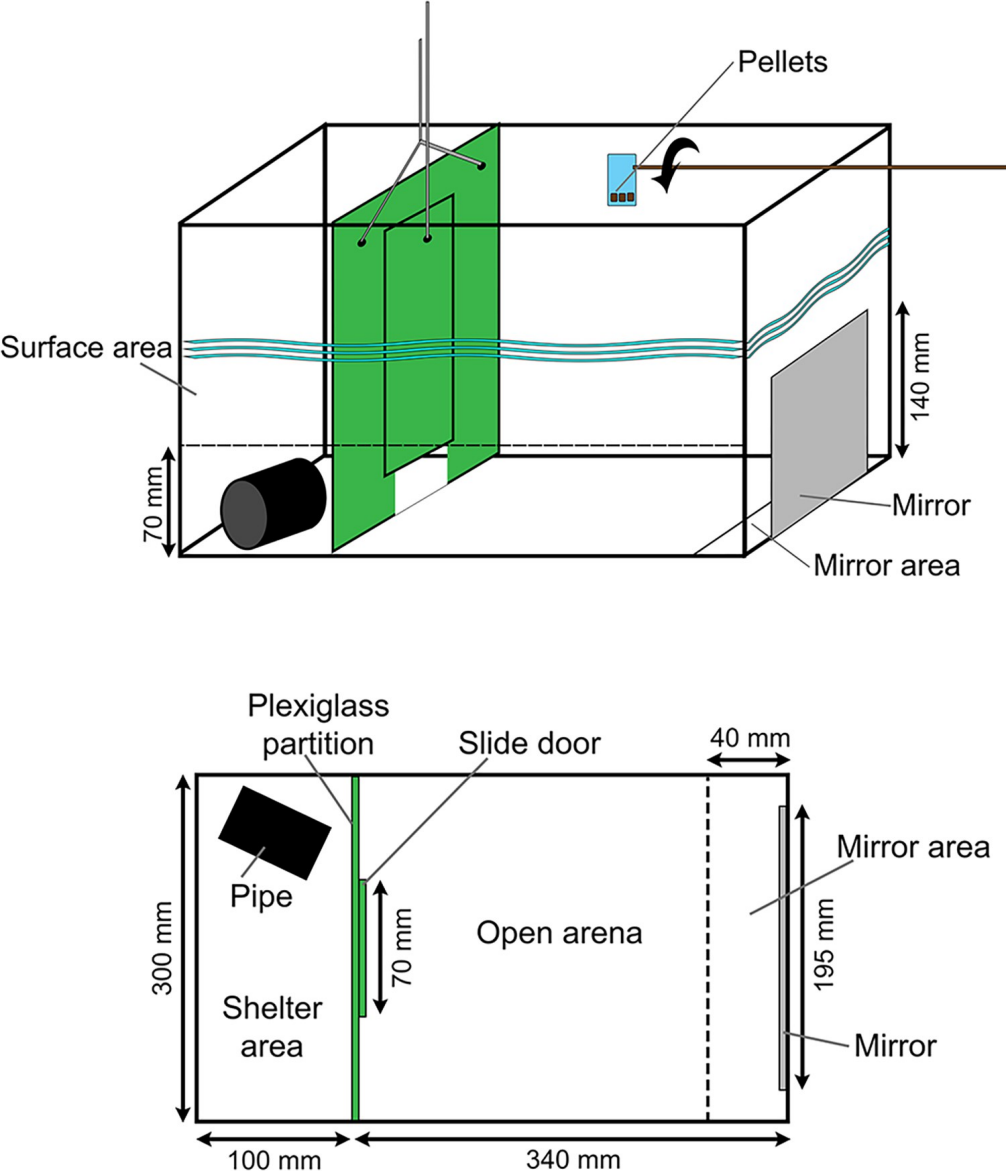
Schematic representation of the experimental tank used for behavioural experiments. The diagram shows the different areas of the tank and their measures. The opaque green plexiglas partition divides the shelter area from the ‘open arena’ area through a sliding door raised remotely by a pulley.

The three context-specific experiments were completed within three 20 L aquaria. Each aquarium was split into two chambers by an opaque plexiglass partition with a sliding door at the bottom (50 x 70 mm) (Fig 1). When the partition was lifted with a pulley system, the fish were able to move between the two compartments. The first chamber (100 x 300 mm) was designated as the ‘shelter’ area (acclimation chamber), with approximately 100 mm of drain-pipe (50 mm radius) providing refuge, with the second chamber being the ‘open arena’ (300 x 340 mm). The ‘open arena’ area was marked vertically at 70 mm from the bottom to enable assessment of the number of times individuals moved through the water column, and had a mirror (140 x 195 mm) on the opposite side of the aquarium to the shelter, covered with an opaque plexiglass sheet so that it could not be seen initially, but could be uncovered using a pulley system. A line on the bottom of the aquarium 40 mm in front of the mirror allowed quantification of mirror approaches. Exterior to the aquarium, a small food scoop enabled food items to be released into the water without disturbing the fish (Fig 1). This design of the aquaria enabled each experiment to be completed sequentially without having to handle or disturb the fish. To further reduce disturbance of fish during the experiments, the aquariums were placed on shelves with their sides and back covered with black fabric and fish responses were recorded with Crosstour cameras (Action Camera CT7000) positioned by one side of the aquaria. The three experimental aquaria were used concomitantly that were set out on shelving in ‘R’ (right), ‘M’ (middle), and ‘L’ (left) positions to receive the same amount of light.

The experimental sequence commenced with three fish being transferred from a holding aquarium and released individually into one of the three experimental aquaria, where it was placed in the shelter area and held for 30 minutes of acclimation. The door was then opened to enable the fish to enter the experimental chamber, with the sequence of experiments being open-field, mirror stimulation, then foraging behaviour (see below for specific information on their designs). Each of these individual experiments lasted 20 minutes, based on preliminary trials where 38 fish were allowed to perform each experiment for 60 minutes, with 84% of the fish always completing the tests in less than 20 minutes. On completion of the foraging behaviour experiment, the fish was removed from the aquarium, weighed and its PIT tag code recorded. A set of experiments was completed in the morning (8:30 to 10:00) and then a new set in the late morning (11:00 to 12:30). Between these experiments, all experimental aquaria had a complete water change. The videos were processed with BORIS software (Behavioral Observation Research Interactive Software, https://www.boris.unito.it).

To test the repeatability of the behaviours, each fish completed the experimental sequence on three occasions. To randomise any behavioural influences resulting from aquarium position (i.e. ‘aquarium effects’), each of the 3 replicates per fish took place in the ‘R’, ‘M’ and ‘L’ aquaria. To minimise handling stress responses, individuals were pair-housed after the first experiment (this facilitated the recapture of the individual for the next experiment and reduced the stress of trying to catch the same individual among a large number of conspecifics) and, to allow for the expression of natural behaviours, the minimum time between replicates for the same fish was 46 hours (maximum 50 hours). All fish were starved for 24 hours before the sequence of experiments started.

### Experimental sequence

After the 30 minutes of experimental acclimation, the Open field test started with the opening of the sliding door, enabling the individual *B*. *barbus* to move between the two chambers of the aquarium (shelter area and open arena, the sliding door kept open during the experiment). Open field experiments have been used in other fish studies to determine the exploratory behaviours of individuals (more exploratory individuals have shorter latency times to leave the shelter area) and their degree of activity (time (s) individuals are actively swimming in the open arena) [30, 33]. During the acclimation and experimental periods, the following behaviours were recorded for each individual fish (with time always recorded in seconds and where Num = frequency, Totaldur = total time duration of the behaviour and Durmean = mean time duration of the behaviour): Acclimation time Active in the shelter (AA-Num, AA-Totaldur and AA-Durmean), Latency to Exit of the shelter area (LE-Totaldur), time Not Active in the Arena (NAA-Num, NAA-Totaldur and NAA-Durmean), Time in the Shelter (TS-Num, TS-Totaldur), Active Time in the Shelter (swimming) (TSA-Num, TSA-Totaldur and TSA-Durmean), Inactive Time in the Shelter (TSI-Num, TSI-Totaldur and TSI-Durmean), and Time in the Surface Area (TSA-Num, TSA-Totaldur and TSA-Durmean) (see Supporting Information, S1 Table for full descriptions). Following the open field experiment, the mirror stimulation test commenced with the lifting of the opaque sheet covering the mirror in the open arena. The opaque plexiglass partition between both chambers was also removed (as fish might have ended the previous experiment there). In this test, sociability is measured as the tendency and time spent by the individual near the mirror [34, 35]. The use of mirror rather than a conspecific was chosen in order to control for variability in the behaviour exhibited by vital stimuli and to be able to standardise the responses of the same individual in successive replicates [35, 36]. This test can also used to determine aggressiveness in individuals (e.g. attacking the mirror image), however during the development of this test no aggressive behaviour was detected. During this test the following behaviours were recorded: Latency to first approach to the Mirror (LM-Totaldur) and Time Near the Mirror (TNM-Num, TNM-Totaldur and TNM-Durmean) (S1 Table). After 20 minutes, that experiment concluded and the foraging behaviour experiment began. Five food items (as pelletised fishmeal of 2 mm diameter) were added to the tank using the external release system (mirror and opaque plexiglass partition remained exposed and removed). Shorter times to approach the food items and a higher number of items consumed were considered as more exploratory and bolder behaviours. During this test the following behaviours were recorded: Latency to first approach to the Pellets (LP-Totaldur), number of Approaches to the Pellets (PPA-Num), number of times fish attempted to eat Pellets (PPE-Num), and number of Pellets Expelled (PPEX-Num). Fish were observed to occasionally take pellets but then expel rather than ingest them; these attempts were considered as foraging behaviour and for subsequent analyses a maximum value of 5 was used for the total number of pellets consumed (PPE-Num2) (S1 Table). Additional behaviours such as "Escape Behaviour" (EB-Num) and “Other Behaviours” (OBNum) were recorded across the whole experimental period (S1 Table).

### Data and statistical analyses

Across the experimental period, 48 individuals were tested, but two individuals died, some videos failed to record properly (9 videos), two individuals were eliminated because human errors were made during the experimental procedure, and some individuals failed to respond to at least one of the experimental replicates (10 individuals). Thus, only individuals who completed at least one of the experiments in each of their three replicates were included in analyses (n = 25). All statistical analyses were then performed using R version 4.1.2 (R Core Team, 2021) within R Studio version 2021.9.2.382 [37].

Two data sets were used in these analyses; the first contained all of the data from the three replicates per fish (‘full data set’) and the second contained the mean values of the three replicates per fish (‘mean data set’). To reduce the number of variables and select those of most relevance to the expressed behaviours, preliminary principal component analysis (PCA; ‘FactoMineR’ package; [38]) was performed on the data of each separate experiment (open field, mirror-image stimulation and foraging behaviour test) for both data sets (‘full data set’ and ‘mean data set’) (S1 Appendix). For each experiment, the two variables with the highest loadings for each dimension with eigenvalues greater than 1, were selected as the representative variables. The representative variables extracted from the ‘full data set’ preliminary PCAs were used to calculate the repeatability of behaviours, whereas the variables obtained from the ‘mean data set’ preliminary PCAs were then used to construct a final PCA from which the proactive-reactive axis (Dimension 1) was extracted. Finally, the relationships between selected variables was assessed by Pearson correlation tests when original variables followed a normal distribution or when a Jhonson transformation resulted in normality of the variables (Shapiro–Wilk test, all p > 0.05).

Repeatability of behaviours was calculated using the variance components obtained from generalised linear mixed-effects models (lmer function; “lme” package, [39]), where the variables selected from the preliminary PCA (‘full data set’) were standardised (to mean = 0 and SD = 1) and used as dependent variables. Fish weight (average final weight across the three replicates, standardised (to mean = 0 and SD = 1)), aquarium position (factor of three levels, as “R”, “M” and “L”), replicate (factor of three levels, replicate 1, 2 and 3) and hour (factor of two levels, time period at which the experiment took place) were the fixed effects, and fish identity was included as a random effect. Repeatability was calculated as the ratio of the among-individual variance on the sum of the among- and within-individual variance [40, 41], with values described as: low repeatability R ≤ 0.2; moderate repeatability R > 0.2 to ≤ 0.4; and high repeatability R ≥ 0.4 [3, 42]. When the model including fish identity showed more parsimony than the model without (lower AIC values), we considered that the analysed behaviour showed significant repeatability.

### Ethical note

The experiment and all regulated procedures were completed under UK Home Office Project Licence P47216841 and following ethical approval by the Animal Welfare and Ethical Review Board of Bournemouth University.

## Results

### Selected behaviours

Across the 25 *B*. *barbus* that completed at least one of the experiments (mean final mass (± SD) 7.74 ± 1.38 g, range 5.00–10.80 g), the 16 studied behaviours (29 variables) showed high variability (S1 Table). Behaviours that were rarely expressed by the fish during experiments, such as escape behaviour, other behaviours and time not active in the arena (86.67–96% of unresponsive fish, S1 Table), were excluded from the PCAs. Only two individuals expressed a high number of escape behaviours, so this was considered a stress symptom behaviour and these fish were excluded from further analyses. There were also three fish that were in the area close to the mirror when the mirror stimulation test started, resulting in their latency time to approaching the mirror being zero, so these individuals were also removed from further analyses. These steps reduced the final data sets to n = 20 individuals.

### Reactive-proactive axis

Preliminary PCAs on the ‘mean data set’ were used to reduce the number of variables and select those of most relevance to the expressed behaviours on the data of each separate experiment (open field, mirror-image stimulation and foraging behaviour test) (S1 Appendix). The open field experiment had eigenvalues above 1 for two dimensions, whereas the mirror-image stimulation and foraging behaviour had eigenvalues of more than 1 in one dimension (S2 Table). A total of seven variables across the experiments had the highest loadings and were selected to perform the final PCA. These variables were three from the open field experiment: time active in acclimation, and latency to exit (dimension 1; eigenvalue 3.40), and mean time inactive in the shelter (dimension 2; eigenvalue 1.48); two from the mirror-image simulation experiment: latency to first mirror approach, and number of mirror approaches (dimension 1, eigenvalue 2.69); and two from foraging behaviour experiment: latency to first pellet approach, and number of pellet approaches (dimension 1, eigenvalue 2.31) (S2 Table).

**Fig 2:**
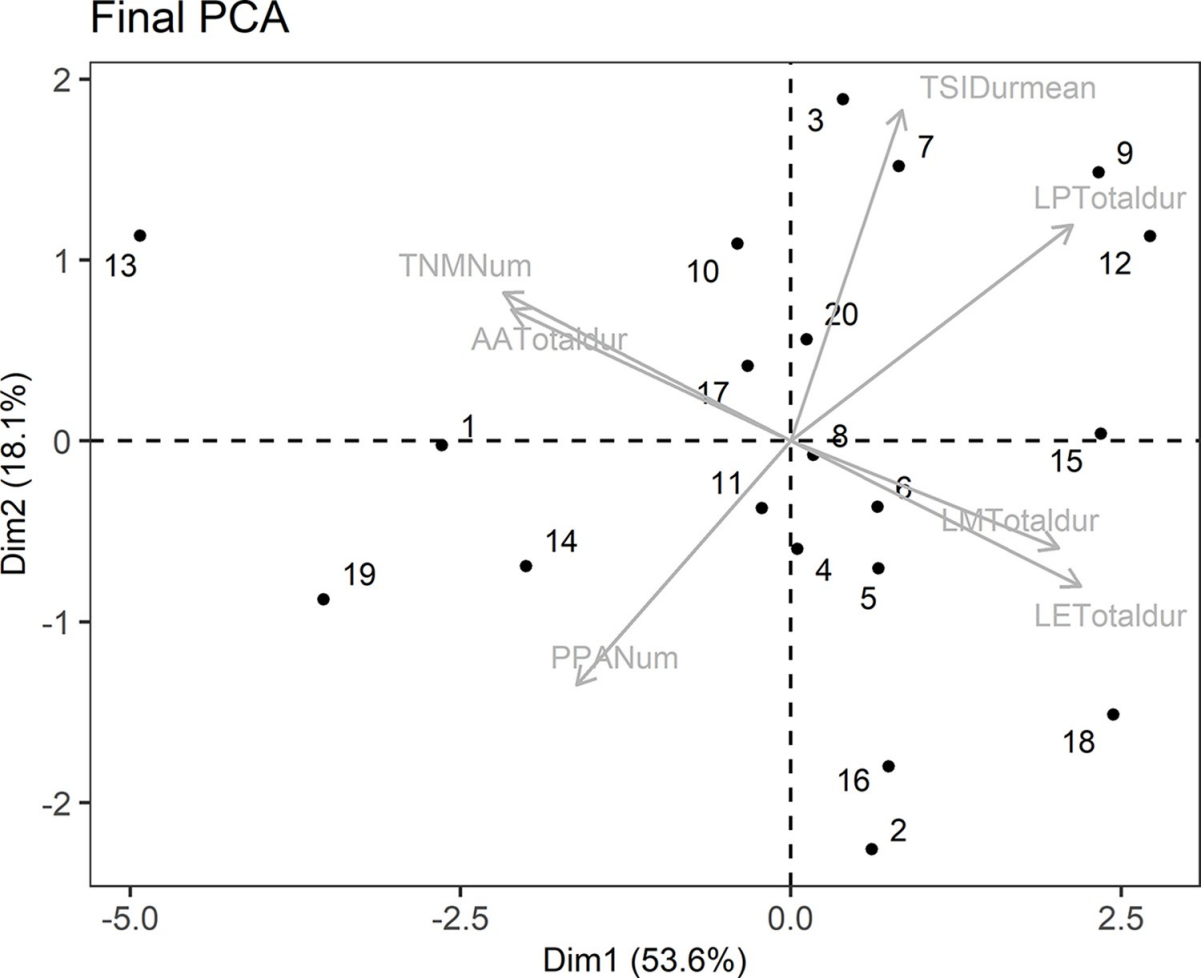
Final principal component analysis of behavioural variables selected from the preliminary PCAs. Individual distribution can be observed along a bold-shy axis (dimension 1).

The final PCA used only the seven variables identified in the preliminary PCAs (Table 1). The first dimension explained 53.6% of the variance and distributed the individuals between higher latency times (higher values of axis 1, representing reactive individuals) and higher time active during the acclimatisation period and number of mirror and pellet approaches (lower values on axis 1, representing proactive individuals) (Fig 2). Individuals that had longer latency times spent less time active during the acclimation period and made fewer approaches to the mirror or the pellets. This axis ranged from -4.93 to 2.72, with most individuals showing a more reactive personality. All variables that had higher loadings in dimension one were significantly related (S1 Fig).

**Table 1:** Principal component results of the selected behavioural variables from the final PCA.

Final PCA mean data set	Dim 1	Dim2	Dim 3
Behavioural variables	Loadings		
**Active in Acclimatation**	**-0.804**	0.276	0.205
**Latency to exit of shelter**	**0.833**	-0.304	-0.276
Mean Inactive Time in shelter	0.319	0.694	0.561
**Latency to first mirror approach**	**0.770**	-0.224	0.391
**Number of mirror approaches**	**-0.827**	0.311	-0.418
**Latency to first pellet approach**	**0.809**	0.454	-0.215
Number of pellet approaches	-0.617	-0.512	0.419
Eigenvalue	3.75	1.27	0.98
Percentage of total variance	53.61%	18.08%	14.02%

Principal component loadings, eigenvalues and percentage of total variance explained for each component. Variables with higher loadings in dimension 1 are marked in bold.

### Repeatability of behaviours

The ‘full data set’ (including the values of the three replicates) was used to study the repeatability of behaviours. First, the number of variables were reduced using preliminary PCAs on the data of each separate experiment (open field, mirror-image stimulation and foraging behaviour test) (following the steps indicated in S1 Appendix). The open field experiment had eigenvalues above 1 for two dimensions, whilst the mirror-image stimulation and foraging behaviour had eigenvalues of more than 1 in one dimension (S2 Table). A total of eight variables with the highest loadings across the three experiments were selected (S2 Table). These were four variables from the open field experiment (latency to exit, active time in the shelter (dimension 1; eigenvalue 3.25); inactive time in the shelter, and number of times inactive in the shelter (dimension 2; eigenvalue 1.93)); two from the mirror-image stimulation experiment (latency to first mirror approach, and number of mirror approaches (dimension 1, eigenvalue 2.49)); and two from the foraging behaviour experiment (latency to first pellets approach, and number of pellets eaten (dimension 1, eigenvalue 2.17)) (S2 Table).

Repeatability was measured on these eight selected variables. Comparison of the models revealed that the variables showing significant repeatability were latency to exit of the shelter area, active time in shelter, number of mirror approaches, latency to first pellet approach and number of pellets eaten (lower values of AIC, S3 Table). Latency to exit of the shelter area, active time in shelter and number of pellets eaten showed high repeatability (values above 0.4) while the remaining variables showed moderate repeatability (values between 0.2 and 0.4) (Table 2). Within-individual variability in behavioural response among replicates can be observed (S2 Fig).

**Table 2:** Variance and repeatability values of the variables selected in the preliminary PCAs.

	Among-individual variance	Within-individual variance	Repeatability
Exploration (open-field test)			
Latency to exit	0.39	0.49	0.44
Active Time in shelter	0.35	0.50	0.41
Boldness (pellet test)			
Latency to first pellet approach	0.30	0.63	0.32
Number of pellets eaten (PPENum2)	0.39	0.46	0.46
Sociability (mirror test)			
Number of mirror approaches	0.26	0.65	0.29

Values of among-individual variance, within-individual variance and repeatability from the random effects of generalised linear mixed-effects models. Behavioural variables selected had the lower AIC values (S3 Table).

## Discussion

It was apparent that there was considerable variation in the measured behaviours among individuals in these *B*. *barbus*, but with their within individual behaviours being relatively consistent. The results revealed the fish were thus expressing phenotypic diversity in their behaviours rather than plasticity at the individual level, with the among-individual behavioural differences being on a proactive-reactive axis, which was consistent with our prediction.

In our study, the measured latency times (to leave the shelter, first mirror approach and first pellet approach) had the highest loading on the dimension 1 in the preliminary PCAs for each experiment (for both the mean and full data sets). These variables have previously been used in other studies on fish personality (as an indicator of the main behaviour described in each experiment), and also by distributing individuals along a proactive-reactive axis or shy-bold continuum [14, 43, 44]. Shorter latency times to leave a shelter to enter a novel environment or to approach prey are found in more proactive individuals and, in addition, a higher number of approaches to conspecifics or to a novel object (mirror and pellets) imply a higher level of pro-activeness [14, 43, 44]. Our results distributed the individuals along the whole axis, confirming the existence of different personalities. More reactive individuals were clustered at the right end of the axis, where latency times were longer, while more proactive fish were distributed at the left end of the axis which showed longer active time during the acclimatisation period and higher number of approaches to the mirror or pellets.

Behavioural syndromes influence the spatial movement dynamics of organisms [14, 22]. Proactive individuals have more explorative behaviours, linked with higher dispersal tendencies or home ranges [14, 45]. In our study, most of the individuals showed a majority reactive personality. Studies on the behaviour and movement of wild *B*. *barbus* in rivers in England and the Czech Republic have indicated approximately 80% of individuals show limited movement (resident component), while the remaining individuals move long distances (mobile component) [25, 28, 46]. Since in general, reactive individuals tend to take fewer risks, express lower locomotor activity, and fewer dispersal and migratory tendencies [47, 48], it appears that these laboratory results might be reflecting to some extent the proportion of personalities in nature. However, some caution is needed here when making this interpretation, given we are working with individuals of different origin (wild vs hatchery), and more research on the behaviour of wild individuals has to be done in order to be able to relate these studies to each other, such as through telemetry-based studies that enable individual behaviours to be measured in wild conditions. In addition, this proportion of individuals being more resident is not always reflected in *B*. *barbus* species in the wild, as environmental conditions and habitat alterations can influence individual mobility [49–51]. Indeed, changes in behavioural responses are influenced and modulated by a range of intrinsic factors, such as genotype, sex and life stage [22, 52, 53], as well as extrinsic factors including habitat complexity, food availability, predator pressure and water temperature [14, 52, 54, 55]. These factors can also influence spatial movement dynamics where, for instance, wild Atlantic cod *Gadus morhua* L. 1758 demonstrated that while all individuals moved and dispersed equally at cold temperatures, reactive individuals tended to reduce their home range as sea temperature increased, while proactive cod increased their rates of dispersal [14].

Animal personality or consistent individual behavioural variation involves a wide range of correlated and consistent behaviours which, if some of them are correlated with each other across individuals at the population level, can be described as related to behavioural syndromes [4, 5]. Within these behavioural syndromes there is high within- and among-individual variability, and these differences are critical for species to cope with environmental challenges. However, for a trait to be a determinant in the survival of an individual or population, it should be consistent over time, i.e. repeatable and, ultimately, heritable [5, 13, 56]. Although behavioural repeatability has been studied by different methods [3, 16], it can be understood as the proportion of behavioural variation that is the result of differences among individuals in the same population [40, 41]. In the present study, latency to exit of the shelter area, active time in shelter and number of pellets eaten showed higher repeatability than other variables. Repeatability of exploratory behaviour (latency to exit of the shelter area) has been demonstrated in other teleost species [57–59]. For example, three different strains of zebrafish *Danio rerio* (Hamilton 1822) showed a range of 0.29–0.71 of repeatability in their exploratory behaviour (measured as stationary time in an open field test) [58]. In juveniles of the convict cichlid *Amatitlania siquia* Schmitter-Soto 2007, repeatability estimates for boldness and exploration behaviour averaged 0.30 and 0.31 respectively [59]. Three-spined sticklebacks *Gasterosteus aculeatus* L. 1758 were also consistent in their foraging behaviour (risk-taking) over time, but this consistency was conditioned by the personalities of the individuals, with bolder fish having a more variable response in their refuge use between trials than shyer ones [60].

Although there are many studies demonstrating the repeatability of many physiological and behavioural traits [3, 16], the level of repeatability varies according to the traits, species or environment conditions [3, 17]. When repeatability is to be quantified, it is necessary to take into account that there are several confounding factors which could alter the results or their interpretation [16]. For instance, ageing or habituation of individuals to laboratory conditions may modify the repeatability response, as organisms undergo a series of metabolic changes as they grow and go through different life stages [16]. Increasing the number of replicates of an experiment offers more robust repeatability estimates, but it could result in individuals becoming habituated to the experiments or learning from one replication to the next. In addition, the origin of study animals (wild or hatchery) also may be a source of repeatability bias [16]. Studies comparing the repeatability degree of behaviours among wild or hatchery individuals showed that exploratory behaviour of zebrafish wild strain was moderately repeatable while selected strains had high repeatability values [58]. Also, repeatability of exploration behaviour within foraging tasks showed that wild brown trout *Salmo trutta* L. 1758 parr were more consistent in their exploration strategy than hatchery individuals [57]. Furthermore, variability in the repeatability of behavioural traits is environmental context dependent [3, 16]. This means that factors such as temperature, oxygen level, water acidification, flow, pollution, parasites or food availability can affect the repeatability response in organisms [16]. Since our study was conducted under standardised and controlled laboratory conditions, it was assumed that environmental fluctuations were sufficiently minimised so that they did not generate a behavioural impact on individuals. All individuals were hatchery reared and with similar sizes, so the confounding factors as collection origin, and differences in body size and ages, were eliminated. The sequence of experiments was the following: open field test, mirror-image stimulating test and foraging test. The order of the experiments could vary the behaviour of individuals, so that individuals may be modulated in their foraging behaviour according to the social context. For example, three-spined sticklebacks were shyer when foraging alone, but became relatively bolder when foraging with more individuals [10]. However, a study on juvenile cod that did consider the order of the experiments found that it had no clear effect on personality traits [61].

Social context also has an important influence on personality and behaviour at the individual level in fish [62]. Some species show a different behavioural response in experiments when housed in groups or individually, with bold individuals also being less plastic in their behavioural response than shy individuals in this context [63–65]. Furthermore, social context also affects the repeatability of behaviours and the level of boldness over time [66, 67]. For example, three-spined stickleback housed solitarily showed greater repeatability in their behaviours and were bolder during the first experiment than fish housed socially [66]. In our study, to reduce the stress of handling and isolation that can be generated in a social species, for the first replicate, individuals were taken from the general tank, where all individuals were housed together. However, for the second and third replicates, individuals were housed in pairs. This was done to make it easier to repeat the experiment and to avoid the stress of chasing the fish in the main tank until to get the individual with the correct PIT tag. This difference in social context could have influenced the variability or repeatability of behaviours. However, this was not always reflected in the response of the individuals during the experiments, as some of the individuals showed similar responses across the three repeats of the experiment. Therefore, caution should be exercised when interpreting the results obtained in personality studies, as we mentioned above, the social context in gregarious species is another factor that could induce behavioural variability [63–66].

To conclude, the results obtained in our study confirm the existence of fish personality along a proactive-reactive axis in *B*. *barbus*, with some of these behaviours showing consistency over time. Further experiments could be carried out to determine whether the repeatability of behaviours is maintained in the long term and if there are differences between proactive and reactive individuals as has been found in other species [58]. In addition, studies about whether these behaviours are correlated with other metabolic or physiologic traits would be important for confirming the existence of pace of life syndromes in the species. It appears that our laboratory results may be reflecting to some extent the proportion of personalities in the field; future research on measuring behavioural traits in the same individuals both in the laboratory and in the field may help to elucidate whether laboratory behavioural experiments reflect behaviours in nature (such as differences in movement behaviour). Since phenotypic diversity in personality is important in how individuals develop in the wild, expanding knowledge about whether behaviours are consistent over time and how personality influences the way individuals relate to their immediate environment (e.g. temperature preference, presence of predators, space movements) could help develop understandings and predictions of how populations of these species will evolve in the new environmental scenarios expected from climate change.

## Supporting information

S1 Appendix Procedure and steps of the preliminary principal component analyses (PCA). (DOC)

S1 Table Description of the behavioural variables. Behaviours analysed in individual European barbel during personality experiments (time in seconds), code, description, mean and range values of the variables and including percentage of fish that did not show the focal behaviour.
(DOCX)

S2 Table Results of the preliminary PCAs. Principal component loadings of behavioural variables, eigenvalues and percentage of total variance explained for each component of preliminary PCAs for the mean data set and full data set. Variables in bold are the highly loaded variables for each component.
(DOC)

S3 Table Comparison of mixed models and linear models for the variables selected in the preliminary PCAs (full data set). Summary and comparison of mixed and linear models and AIC values. Fixed effect variables: weight (average final weight of the three replicates, scaled), replicate (factor of three levels, replicate 1, 2 and 3; Day 2 and Day 3), hour (factor of two levels, first or second hour at which the experiment took place; Hour 2) and aquarium position (factor of three levels, as “R”, “M” and “L”; Aquarium 2 and Aquarium 3).
(DOC)

S1 Fig Pearson correlation between the variables selected in the final PCA. (DOCX)

S2 Fig Graphs showing the individual variation across the replicates. Within-individual variation across the three replicates of individuals ranked according to their boldness gradient. Variables shown are those that were found to be most representative in the preliminary PCAs from the full data set.
(DOCX)
