# Supplementary material for: Individual phenotypic variability in the behaviour of an aggregative riverine fish is structured along a reactive-proactive axis
Source: PLoS One. 2024 Nov 20;19(11):e0312187. doi: 10.1371/journal.pone.0312187 (PMC11578482; doi:10.1371/journal.pone.0312187)
Supplement: S1 Appendix — (DOC) [file pone.0312187.s001.doc]

**Supplementary Information:**

**Individual phenotypic variability in the behaviour of an aggregative riverine fish is structured along a reactive-proactive axis**

Fatima Amat-Trigo, Demetra Andreou, Phillipa K. Gillingham and J. Robert Britton

**S1 Appendix. Procedure and steps of the Preliminary Principal Component Analyses (PCA)**

In order to select the most representative behaviours and reduce the number of variables in each of the tests performed (acclimatation period and open-field test 18 variables, mirror-image stimulation test 5 variables and foraging behaviour test 5 variables) (S1 Table) we performed principal component analysis for each of the tests separately.

First, we studied the correlation of the variables (correlation matrix) to eliminate those that were redundant for the analysis (we eliminated variables with a correlation value higher than 0.80). The second step was to apply the Kaiser-Meyer-Olkin (KMO) test, which indicates the suitability of the data for factor analysis. Factor analysis was only performed when KMO values above 0.60 were obtained. If, after eliminating variables with a high correlation, the KMO test value was lower than 0.60, we eliminated one by one the variables that showed lower measures of sampling adequacy (MSA), until we obtained a set of variables in which the KMO value was higher than 0.60. When the set of variables was adequate, we used the PCA function of the "FactoMineR" package (note that the data are automatically standardised with this function) to perform the principal component analysis. We retained the components with eigenvalues greater than 1 and selected the two variables with the highest loadings in each of the components for the following analyses.

We performed these preliminary PCAs for both data sets, the ‘full data set’ (containing the data of the three replicates) and the ‘mean data set’ (containing the mean value of the replicates) (S2 Table).
