# Supplementary material for: Individual phenotypic variability in the behaviour of an aggregative riverine fish is structured along a reactive-proactive axis
Source: PLoS One. 2024 Nov 20;19(11):e0312187. doi: 10.1371/journal.pone.0312187 (PMC11578482; doi:10.1371/journal.pone.0312187)
Supplement: S1 Table — Behaviours analysed in individual European barbel during personality experiments (time in seconds), code, description, mean and range values of the variables and including percentage of fish that did not show the focal behaviour. (DOCX) [file pone.0312187.s002.docx]

**Supplementary Information:**

**Individual phenotypic variability in the behaviour of an aggregative riverine fish is structured along a reactive-proactive axis**

Fatima Amat-Trigo, Demetra Andreou, Phillipa K. Gillingham and J. Robert Britton

**S1 Table.** Behaviours analyzed in individual European barbel during personality experiments (time in seconds), code, description, mean and range values of the variables and including percentage of fish that did not show the focal behaviour.

| **Behaviour** | **Variables** | **Code** | **Description** | **Mean** | **Range** | **Unresponsive** |
| --- | --- | --- | --- | --- | --- | --- |
| Acclimation Time active in the shelter | Total number of occurrences | AA-Num | Number of times the individual moves during the acclimation period | 11.53 | 0.0 - 29.00 | 2.67% |
|  | Total duration (s) | AA-Totaldur | Total time the individual is active during the acclimation period | 608.63 | 0.0 - 1692.69 |  |
|  | Duration mean (s) | AA-Durmean | Average length of time the individual is active during the acclimation period (AATotaldur/ AANum) | 58.98 | 0.0 - 310.43 |  |
| Latency to exit of the shelter area | Total duration (s) | LE-Totaldur | Total time it takes for the individual to leave the shelter area | 724.28 | 2.65- 1313.20 | 25.33% |
| Time no-active in the arena | Total number of occurrences | NAA-Num | Number of times the individual remains immobile in the open arena | 0.41 | 0.0 - 9.00 | 86.67% |
|  | Total duration (s) | NAA-Totaldur | Total time the individual remains immobile in the open arena | 12.26 | 0.0 - 368.28 |  |
|  | Duration mean (s) | NAA-Durmean | Average length of time the individual remains immobile in the open arena (NAATotaldur/ NAANum) | 3.30 | 0.0 - 73.66 |  |
| Time in shelter | Total number of occurrences | TS-Num | Number of times the individual re-enters the shelter area after leaving it the first time. | 4.40 | 0.0 - 18.00 | 26.67% |
|  | Total duration (s) | TS-Totaldur | Total time in the shelter area | 320.64 | 0.0 - 1010.36 |  |
| Active Time in shelter | Total number of occurrences | TSA-Num | Number of times the individual is active (swimming) in shelter area | 5.89 | 0.0 - 20.00 | 28.00% |
|  | Total duration (s) | TSA-Totaldur | Total time active in the shelter area | 219.72 | 0.0 - 992.60 |  |
|  | Duration mean (s) | TSA-Durmean | Average length of time the individual is active in the shelter area (TSATotaldur/ TSANum) | 27.20 | 0.0 - 245.61 |  |
| Inactive Time in shelter | Total number of occurrences | TSI-Num | Number of times the individual is inactive in shelter area | 1.93 | 0.0 - 12.00 | 40.00% |
|  | Total duration (s) | TSI-Totaldur | Total time inactive in the shelter area | 100.93 | 0.0 - 619.20 |  |
|  | Duration mean (s) | TSI-Durmean | Average length of time the individual is inactive in the shelter area (TSITotaldur/ TSINum) | 29.61 | 0.0 - 141.05 |  |
| Time surface area | Total number of occurrences | TSA-Num | Number of times the individual swims near the surface | 10.15 | 0.0 - 94.00 | 44.00% |
|  | Total duration (s) | TSA-Totaldur | Total time near the surface | 32.58 | 0.0 - 256.03 |  |
|  | Duration mean (s) | TSA-Durmean | Average length of time the individual swims near the surface (ESATotaldur/ ESANum) | 1.65 | 0.0 - 5.695 |  |
| Escape behaviour | Total number of occurrences | EB-Num | Number of times the individual attempts to jump out of the aquarium. | 0.75 | 0.0 - 36.00 | 92.00% |
| Latency to first approach mirror | Total duration (s) | LM-Totaldur | Total time it takes for the individual to approach to the mirror the first time | 628.95 | 1.505 - 1345.49 | 24.00% |
| Time near the mirror | Total number of occurrences | TNM-Num | Number of times the individual is near the mirror | 9.76 | 0.0 - 39.00 | 24.00% |
|  | Total duration (s) | TNM-Totaldur | Total time near the mirror | 300.52 | 0.0 - 1071.32 |  |
|  | Duration mean (s) | TNM-Durmean | Average length of time the individual is near the mirror (TNMTotaldur/ TNMNum) | 24.90 | 0.0 - 152.22 |  |
| Other behaviours | Total number of occurrences | OB-Num | Number of times the individual has any of these behaviours: biting, head down, backward swimming | 0.12 | 0.0 - 5.00 | 96.00% |
| Latency to first approach Pellet | Total number of occurrences | LP-Totaldur | Total time it takes for the individual to approach to the pellets the first time | 473.43 | 0.749 - 1284.81 | 13.33% |
| Pellet approaches | Total number of occurrences | PPA-Num | Number of times the individual approaches to the pellets | 3.20 | 0.0 - 14.00 | 13.33% |
| Foraging behaviour | Total number of occurrences | PPE-Num | Number of times the individual tries to eat the pellets | 5.08 | 0.0 - 27.00 | 14.67% |
| Pellet expelled | Total number of occurrences | PPEX-Num | Number of times the individual expels to the pellets | 2.61 | 0.0 - 27.00 | 60.00% |
| Pellet eaten | Total number of occurrences | PPE-Num2 | Number of foraging behaviours with a maximum of 5 (total number of pellets offered) | 3.587 | 0.0 - 5.00 | 14.67% |
