# Supplementary material for: Individual phenotypic variability in the behaviour of an aggregative riverine fish is structured along a reactive-proactive axis
Source: PLoS One. 2024 Nov 20;19(11):e0312187. doi: 10.1371/journal.pone.0312187 (PMC11578482; doi:10.1371/journal.pone.0312187)
Supplement: S2 Table — Principal component loadings of behavioural variables, eigenvalues and percentage of total variance explained for each component of preliminary PCAs for the mean data set and full data set. Variables in bold are the highly loaded variables for each component. (DOC) [file pone.0312187.s003.doc]

**Supplementary Information:**

**Individual phenotypic variability in the behaviour of an aggregative riverine fish is structured along a reactive-proactive axis**

Fatima Amat-Trigo, Demetra Andreou, Phillipa K. Gillingham and J. Robert Britton

**S2 Table.** **Results of the preliminary PCAs.** Principal component loadings of behavioural variables, eigenvalues and percentage of total variance explained for each component of preliminary PCAs for the mean data set and full data set. Variables in bold are the highly loaded variables for each component.

| **Preliminary PCA mean data set** | | | | |  | **Preliminary PCA full data set** | | | | |
| --- | --- | --- | --- | --- | --- | --- | --- | --- | --- | --- |
| PC exploration (open-field test) | | | Dim 1 | Dim 2 |  | PC exploration (open-field test) | | | Dim 1 | Dim 2 |
|  | Behavioural variables | | Loadings | |  |  | Behavioural variables | | Loadings | |
|  |  | **Active in Acclimatation** | **0.892** | -0.211 |  |  |  | Active in Acclimatation | 0.820 | -0.219 |
|  |  | **Latency to exit** | **-0.934** | 0.104 |  |  |  | **Latency to exit** | **-0.959** | -0.170 |
|  |  | Mean Active Time in shelter | 0.723 | 0.438 |  |  |  | **Active Time in shelter** | **0.916** | -0.086 |
|  |  | **Mean Inactive Time in shelter** | **0.122** | **0.960** |  |  |  | **Inactive Time in shelter** | 0.176 | **0.935** |
|  |  | Time surface area | 0.865 | -0.439 |  |  |  | **Number of times inactive in shelter** | 0.272 | **0.912** |
|  |  | Mean Time surface area | 0.668 | 0.345 |  |  |  | Time surface area | 0.848 | -0.373 |
|  | Eigenvalue | | 3.40 | 1.48 |  |  | Eigenvalue | | 3.25 | 1.93 |
|  |  | Percentage of total variance | 56.55 | 24.68 |  |  |  | Percentage of total variance | 54.23 | 32.17 |
| PC boldness (foraging behaviour test) | | | Dim 1 | Dim 2 |  | PC boldness (foraging behaviour test) | | | Dim 1 | Dim 2 |
|  | Behavioural variables | | Loadings | |  |  | Behavioural variables | | Loadings | |
|  |  | **Latency to first pellet approach** | **-0.914** | 0.122 |  |  |  | **Latency to first pellet approach** | **-0.886** | 0.121 |
|  |  | **Number of approaches** | **0.878** | -0.382 |  |  |  | Number of approaches | 0.816 | 0.56 |
|  |  | Number of pellets eaten (PPENum2) | 0.837 | 0.535 |  |  |  | **Number of pellets eaten (PPENum2)** | **0.850** | -0.411 |
|  | Eigenvalue | | 2.31 | 0.45 |  |  | Eigenvalue | | 2.17 | 0.50 |
|  |  | Percentage of total variance | 76.87 | 14.91 |  |  |  | Percentage of total variance | 72.42 | 16.58 |
| PC sociability (mirror-image stimulation test) | | | Dim 1 | Dim 2 |  | PC sociability (mirror-image stimulation test) | | | Dim 1 | Dim 2 |
|  | Behavioural variables | | Loadings | |  |  | Behavioural variables | | Loadings | |
|  |  | **Latency to first mirror approach** | **-0.947** | 0.155 |  |  |  | **Latency to first mirror approach** | **-0.914** | 0.273 |
|  |  | **Number of approaches** | **0.938** | -0.244 |  |  |  | **Number of approaches** | **0.921** | -0.157 |
|  |  | Time in the mirror area | 0.910 | 0.412 |  |  |  | Time in the mirror area | 0.896 | 0.439 |
|  | Eigenvalue | | 2.61 | 0.25 |  |  | Eigenvalue | | 2.49 | 0.29 |
|  |  | Percentage of total variance | 86.86 | 8.43 |  |  |  | Percentage of total variance | 82.86 | 9.73 |
