# Supplementary material for: Individual phenotypic variability in the behaviour of an aggregative riverine fish is structured along a reactive-proactive axis
Source: PLoS One. 2024 Nov 20;19(11):e0312187. doi: 10.1371/journal.pone.0312187 (PMC11578482; doi:10.1371/journal.pone.0312187)
Supplement: S3 Table — Summary and comparison of mixed and linear models and AIC values. Fixed effect variables: weight (average final weight of the three replicates, scaled), replicate (factor of three levels, replicate 1, 2 and 3; Day 2 and Day 3), hour (factor of two levels, first or second hour at which the experiment took place; Hour 2) and aquarium position (factor of three levels, as “R”, “M” and “L”; Aquarium 2 and Aquarium 3). (DOC) [file pone.0312187.s004.doc]

**Supplementary Information:**

**Individual phenotypic variability in the behaviour of an aggregative riverine fish is structured along a reactive-proactive axis**

Fatima Amat-Trigo, Demetra Andreou, Phillipa K. Gillingham and J. Robert Britton

**S3 Table. Comparison of mixed models and linear models for the variables selected in the preliminary PCAs (full data set)**. Summary and comparison of mixed and linear models and AIC values. Fixed effect variables: weight (average final weight of the three replicates, scaled), replicate (factor of three levels, replicate 1, 2 and 3; Day 2 and Day 3), hour (factor of two levels, first or second hour at which the experiment took place; Hour 2) and aquarium position (factor of three levels, as “R”, “M” and “L”; Aquarium 2 and Aquarium 3).

| **Response variable** | **Mixed-Model**  **components** | **Estimate** | **SE** | **DF** | **p-value** | **AIC** | **Linear-Model**  **components** | **Estimate** | **SE** | **DF** | **p-value** | **AIC** |
| --- | --- | --- | --- | --- | --- | --- | --- | --- | --- | --- | --- | --- |
| Latency to exit | Fixed effects |  |  |  |  | 169.53 |  |  |  | 53 | 0.372 | 178.19 |
|  | Intercept | 0.15 | 0.26 | 58.37 | 0.574 |  | Intercept | 0.19 | 0.31 |  | 0.552 |  |
|  | Body weight | 0.18 | 0.17 | 23.19 | 0.289 |  | Body weight | 0.18 | 0.13 |  | 0.171 |  |
|  | Day 2 | 0.35 | 0.22 | 40.51 | 0.125 |  | Day 2 | 0.35 | 0.32 |  | 0.271 |  |
|  | Day 3 | -0.15 | 0.22 | 40.35 | 0.503 |  | Day 3 | -0.16 | 0.32 |  | 0.623 |  |
|  | Hour 2 | -0.26 | 0.21 | 46.37 | 0.220 |  | Hour 2 | -0.33 | 0.27 |  | 0.228 |  |
|  | Aquarium 2 | -0.04 | 0.23 | 40.41 | 0.874 |  | Aquarium 2 | -0.02 | 0.32 |  | 0.946 |  |
|  | Aquarium 3 | -0.18 | 0.22 | 40.17 | 0.419 |  | Aquarium 3 | -0.19 | 0.32 |  | 0.558 |  |
|  | Random variance |  |  |  |  |  |  |  |  |  |  |  |
|  | Intercept (among-individual)  Residual (within-individual) | 0.39  0.49 |  |  |  |  |  |  |  |  |  |  |
|  | Repeatability | 0.44 |  |  |  |  |  |  |  |  |  |  |
|  | R^2^ marginal^*^ | 0.10 |  |  |  |  | R^2^ | 0.11 |  |  |  |  |
| Active Time in shelter | Fixed effects |  |  |  |  | 169.34 |  |  |  | 53 | 0.207 | 176.03 |
|  | Intercept | -0.17 | 0.26 | 59.09 | 0.522 |  | Intercept | -0.23 | 0.31 |  | 0.455 |  |
|  | Body weight | -0.11 | 0.16 | 22.50 | 0.493 |  | Body weight | -0.14 | 0.13 |  | 0.276 |  |
|  | Day 2 | -0.47 | 0.23 | 40.02 | **0.046** |  | Day 2 | -0.47 | 0.31 |  | 0.135 |  |
|  | Day 3 | -0.18 | 0.23 | 39.88 | 0.425 |  | Day 3 | -0.17 | 0.31 |  | 0.576 |  |
|  | Hour 2 | 0.36 | 0.21 | 46.64 | 0.091 |  | Hour 2 | 0.48 | 0.27 |  | 0.077 |  |
|  | Aquarium 2 | 0.34 | 0.23 | 39.97 | 0.150 |  | Aquarium 2 | 0.32 | 0.32 |  | 0.322 |  |
|  | Aquarium 3 | 0.22 | 0.23 | 39.71 | 0.337 |  | Aquarium 3 | 0.23 | 0.31 |  | 0.464 |  |
|  | Random variance |  |  |  |  |  |  |  |  |  |  |  |
|  | Intercept (among-individual)  Residual (within-individual) | 0.35  0.50 |  |  |  |  |  |  |  |  |  |  |
|  | Repeatability | 0.41 |  |  |  |  |  |  |  |  |  |  |
|  | R^2^ marginal^*^ | 0.11 |  |  |  |  | R^2^ | 0.14 |  |  |  |  |
| Number of times inactive in shelter | Fixed effects |  |  |  |  | 180.71 |  |  |  | 53 | 0.536 | 179.74 |
|  | Intercept | 0.01 | 0.29 | 58.63 | 0.971 |  | Intercept | 0.02 | 0.32 |  | 0.948 |  |
|  | Body weight | -0.19 | 0.14 | 22.03 | 0.182 |  | Body weight | -0.19 | 0.13 |  | 0.165 |  |
|  | Day 2 | -0.05 | 0.28 | 40.16 | 0.872 |  | Day 2 | -0.04 | 0.32 |  | 0.891 |  |
|  | Day 3 | 0.33 | 0.28 | 40.16 | 0.237 |  | Day 3 | -0.33 | 0.32 |  | 0.301 |  |
|  | Hour 2 | 0.08 | 0.25 | 53.05 | 0.765 |  | Hour 2 | 0.06 | 0.28 |  | 0.841 |  |
|  | Aquarium 2 | -0.36 | 0.29 | 40.44 | 0.211 |  | Aquarium 2 | -0.36 | 0.33 |  | 0.276 |  |
|  | Aquarium 3 | -0.09 | 0.28 | 40.03 | 0.764 |  | Aquarium 3 | -0.09 | 0.32 |  | 0.789 |  |
|  | Random variance |  |  |  |  |  |  |  |  |  |  |  |
|  | Intercept (among-individual)  Residual (within-individual) | 0.12  0.78 |  |  |  |  |  |  |  |  |  |  |
|  | Repeatability | 0.13 |  |  |  |  |  |  |  |  |  |  |
|  | R^2^ marginal^*^ | 0.09 |  |  |  |  | R^2^ | 0.09 |  |  |  |  |
| Inactive Time in shelter | Fixed effects |  |  |  |  | 179.64 |  |  |  | 53 | 0.322 | 177.64 |
|  | Intercept | 0.01 | 0.29 | 60.00 | 0.984 |  | Intercept | 0.01 | 0.31 |  | 0.985 |  |
|  | Body weight | -0.18 | 0.12 | 60.00 | 0.151 |  | Body weight | -0.18 | 0.13 |  | 0.178 |  |
|  | Day 2 | 0.09 | 0.30 | 60.00 | 0.765 |  | Day 2 | 0.09 | 0.32 |  | 0.779 |  |
|  | Day 3 | 0.53 | 0.30 | 60.00 | 0.083 |  | Day 3 | 0.53 | 0.32 |  | 0.103 |  |
|  | Hour 2 | 0.00 | 0.25 | 60.00 | 0.991 |  | Hour 2 | 0.00 | 0.27 |  | 0.992 |  |
|  | Aquarium 2 | -0.46 | 0.30 | 60.00 | 0.132 |  | Aquarium 2 | -0.46 | 0.32 |  | 0.157 |  |
|  | Aquarium 3 | -0.18 | 0.30 | 60.00 | 0.556 |  | Aquarium 3 | -0.18 | 0.32 |  | 0.580 |  |
|  | Random variance |  |  |  |  |  |  |  |  |  |  |  |
|  | Intercept (among-individual)  Residual (within-individual) | 0.00  0.87 |  |  |  |  |  |  |  |  |  |  |
|  | Repeatability | 0 |  |  |  |  |  |  |  |  |  |  |
|  | R^2^ marginal* | 0.12 |  |  |  |  | R^2^ | 0.12 |  |  |  |  |
| Latency to first mirror approach | Fixed effects |  |  |  |  | 180.76 |  |  |  | 53 | 0.449 | 178.96 |
|  | Intercept | -.019 | 0.29 | 57.48 | 0.518 |  | Intercept | -0.19 | 0.31 |  | 0.548 |  |
|  | Body weight | 0.15 | 0.13 | 22.00 | 0.277 |  | Body weight | 0.15 | 0.13 |  | 0.263 |  |
|  | Day 2 | 0.38 | 0.29 | 40.17 | 0.203 |  | Day 2 | 0.38 | 0.32 |  | 0.242 |  |
|  | Day 3 | 0.36 | 0.29 | 40.19 | 0.225 |  | Day 3 | 0.36 | 0.32 |  | 0.265 |  |
|  | Hour 2 | -0.07 | 0.25 | 55.19 | 0.794 |  | Hour 2 | -0.07 | 0.27 |  | 0.810 |  |
|  | Aquarium 2 | 0.22 | 0.30 | 40.54 | 0.458 |  | Aquarium 2 | 0.22 | 0.32 |  | 0.498 |  |
|  | Aquarium 3 | -0.28 | 0.29 | 40.07 | 0.340 |  | Aquarium 3 | -0.28 | 0.32 |  | 0.382 |  |
|  | Random variance |  |  |  |  |  |  |  |  |  |  |  |
|  | Intercept (among-individual)  Residual (within-individual) | 0.05  0.83 |  |  |  |  |  |  |  |  |  |  |
|  | Repeatability | 0.06 |  |  |  |  |  |  |  |  |  |  |
|  | R^2^ marginal* | 0.10 |  |  |  |  | R^2^ | 0.10 |  |  |  |  |
| Number of approaches to the mirror | Fixed effects |  |  |  |  | 178.03 |  |  |  | 53 | 0.610 | 180.36 |
|  | Intercept | 0.14 | 0.28 | 59.98 | 0.617 |  | Intercept | 0.13 | 0.32 |  | 0.674 |  |
|  | Body weight | -0.16 | 0.16 | 22.49 | 0.318 |  | Body weight | -0.16 | 0.14 |  | 0.241 |  |
|  | Day 2 | -0.19 | 0.26 | 40.39 | 0.476 |  | Day 2 | -0.19 | 0.32 |  | 0.568 |  |
|  | Day 3 | -0.33 | 0.26 | 40.32 | 0.213 |  | Day 3 | -0.32 | 0.32 |  | 0.319 |  |
|  | Hour 2 | 0.19 | 0.23 | 49.52 | 0.418 |  | Hour 2 | 0.21 | 0.28 |  | 0.462 |  |
|  | Aquarium 2 | -0.27 | 0.26 | 40.49 | 0.316 |  | Aquarium 2 | -0.27 | 0.33 |  | 0.417 |  |
|  | Aquarium 3 | 0.4 | 0.26 | 40.17 | 0.880 |  | Aquarium 3 | 0.04 | 0.32 |  | 0.902 |  |
|  | Random variance |  |  |  |  |  |  |  |  |  |  |  |
|  | Intercept (among-individual)  Residual (within-individual) | 0.26  0.65 |  |  |  |  |  |  |  |  |  |  |
|  | Repeatability | 0.29 |  |  |  |  |  |  |  |  |  |  |
|  | R^2^ marginal* | 0.08 |  |  |  |  | R^2^ | 0.08 |  |  |  |  |
| Latency to first pellet approach | Fixed effects |  |  |  |  | 178.11 |  |  |  | 53 | 0.792 | 181.84 |
|  | Intercept | -0.03 | 0.28 | 59.96 | 0.909 |  | Intercept | -0.01 | 0.32 |  | 0.975 |  |
|  | Body weight | -0.01 | 0.16 | 22.60 | 0.940 |  | Body weight | -0.01 | 0.14 |  | 0.959 |  |
|  | Day 2 | 0.51 | 0.25 | 40.38 | 0.052 |  | Day 2 | 0.51 | 0.33 |  | 0.126 |  |
|  | Day 3 | 0.19 | 0.25 | 40.29 | 0.441 |  | Day 3 | 0.19 | 0.33 |  | 0.556 |  |
|  | Hour 2 | -0.05 | 0.23 | 48.56 | 0.830 |  | Hour 2 | -0.09 | 0.28 |  | 0.741 |  |
|  | Aquarium 2 | -0.27 | 0.26 | 40.43 | 0.292 |  | Aquarium 2 | -0.27 | 0.33 |  | 0.426 |  |
|  | Aquarium 3 | -0.25 | 0.25 | 40.14 | 0.327 |  | Aquarium 3 | -0.25 | 0.33 |  | 0.441 |  |
|  | Random variance |  |  |  |  |  |  |  |  |  |  |  |
|  | Intercept (among-individual)  Residual (within-individual) | 0.30  0.63 |  |  |  |  |  |  |  |  |  |  |
|  | Repeatability | 0.32 |  |  |  |  |  |  |  |  |  |  |
|  | R^2^ marginal* | 0.05 |  |  |  |  | R^2^ | 0.05 |  |  |  |  |
| Number of pellets eaten (PPENum2) | Fixed effects |  |  |  |  | 167.27 |  |  |  | 53 | 0.242 | 176.59 |
|  | Intercept | 0.45 | 0.26 | 58.01 | 0.090 |  | Intercept | 0.41 | 0.31 |  | 0.185 |  |
|  | Body weight | 0.13 | 0.16 | 23.23 | 0.452 |  | Body weight | 0.11 | 0.13 |  | 0.413 |  |
|  | Day 2 | -0.67 | 0.22 | 40.48 | **0.004** |  | Day 2 | -0.67 | 0.31 |  | **0.037** |  |
|  | Day 3 | -0.77 | 0.22 | 40.32 | **0.001** |  | Day 3 | -0.76 | 0.31 |  | **0.019** |  |
|  | Hour 2 | -0.05 | 0.20 | 46.12 | 0.818 |  | Hour 2 | 0.02 | 0.27 |  | 0.952 |  |
|  | Aquarium 2 | 0.21 | 0.22 | 40.37 | 0.351 |  | Aquarium 2 | 0.20 | 0.32 |  | 0.537 |  |
|  | Aquarium 3 | -0.04 | 0.22 | 40.14 | 0.860 |  | Aquarium 3 | -0.03 | 0.31 |  | 0.916 |  |
|  | Random variance |  |  |  |  |  |  |  |  |  |  |  |
|  | Intercept (among-individual)  Residual (within-individual) | 0.39  0.46 |  |  |  |  |  |  |  |  |  |  |
|  | Repeatability | 0.46 |  |  |  |  |  |  |  |  |  |  |
|  | R^2^ marginal* | 0.14 |  |  |  |  | R^2^ | 0.14 |  |  |  |  |
