# Supplementary material for: Individual phenotypic variability in the behaviour of an aggregative riverine fish is structured along a reactive-proactive axis
Source: PLoS One. 2024 Nov 20;19(11):e0312187. doi: 10.1371/journal.pone.0312187 (PMC11578482; doi:10.1371/journal.pone.0312187)

**Supplementary Information:**

**Individual phenotypic variability in the behaviour of an aggregative riverine fish is structured along a reactive-proactive axis**

Fatima Amat-Trigo, Demetra Andreou, Phillipa K. Gillingham and J. Robert Britton

**S1 Fig. Pearson correlation between the variables selected in the Final PCA**. *** p < 0.001; ** p < 0.01; * p < 0.05.


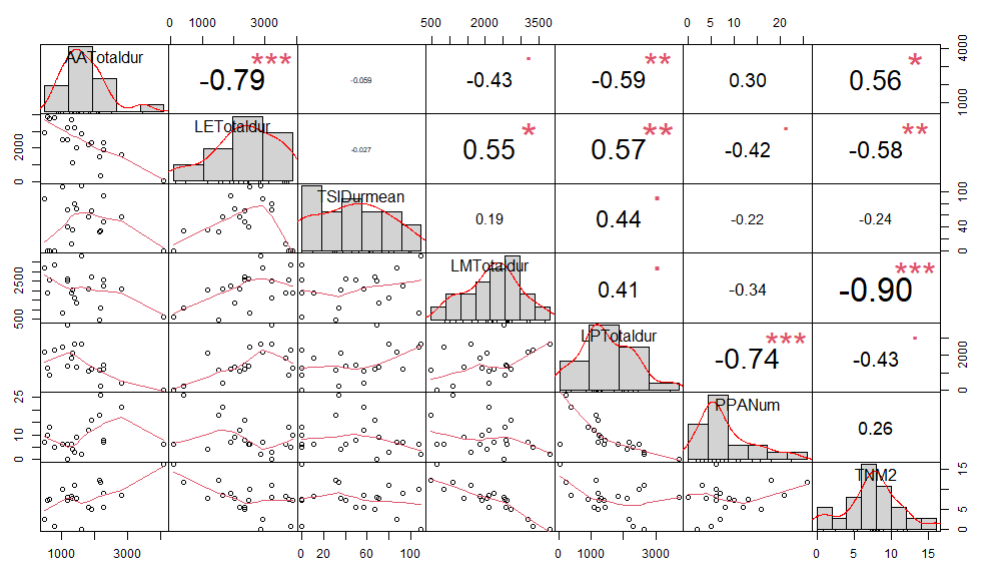

Supplement: S1 Fig — (DOCX) [file pone.0312187.s005.docx]
