# Supplementary material for: Individual phenotypic variability in the behaviour of an aggregative riverine fish is structured along a reactive-proactive axis
Source: PLoS One. 2024 Nov 20;19(11):e0312187. doi: 10.1371/journal.pone.0312187 (PMC11578482; doi:10.1371/journal.pone.0312187)

**Supplementary Information:**

**Individual phenotypic variability in the behaviour of an aggregative riverine fish is structured along a reactive-proactive axis**

Fatima Amat-Trigo, Demetra Andreou, Phillipa K. Gillingham and J. Robert Britton

**S2 Fig. Intra-individual variation across the three replicates of individuals ranked according to their position on the proactive-reactive axis.** Variables shown are those that were found to be most representative in the preliminary PCAs from the full data set.


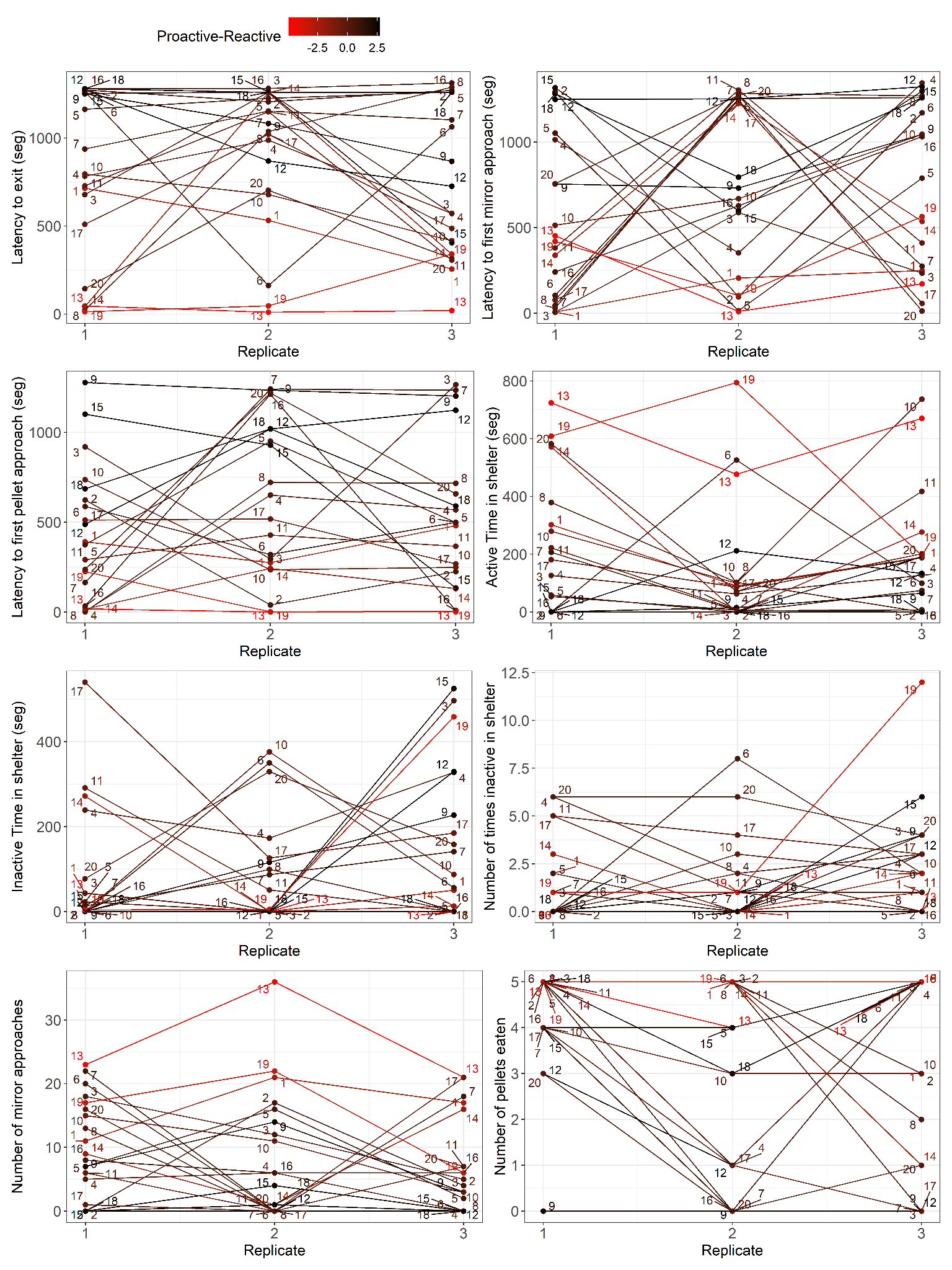

Supplement: S2 Fig — Within-individual variation across the three replicates of individuals ranked according to their boldness gradient. Variables shown are those that were found to be most representative in the preliminary PCAs from the full data set. (DOCX) [file pone.0312187.s006.docx]
